# Supplementary material for: C﻿ommunicating doctors’ consensus persistently increases COVID-19 vaccinations
Source: Nature. 2022 Jun 1;606(7914):542–9. doi: 10.1038/s41586-022-04805-y (PMC9200639; doi:10.1038/s41586-022-04805-y)
Supplement: Supplementary file 1 — This Supplementary Information file contains the following sections: 1. Supplementary Figure 1; 2. Supplementary Tables 1–9; 3. Supplementary Methods; 4. Populated Pre-Analysis Plan. [file 41586_2022_4805_MOESM1_ESM.pdf]

nature portfolio

https://doi.org/10.1038/s41586-022-04805-y

Supplementary information

Communicating doctors' consensus persistently increases COVID-19 vaccinations

In the format provided by the authors and unedited

Supplementary Information

Communicating doctors' consensus persistently increases Covid-19 vaccinations

Vojtěch Bartoš*, Michal Bauer, Jana Cahlíková, Julie Chytilová

*Correspondence to: vojtech.bartos@econ.lmu.de

Contents

1. Supplementary Figure 1 **page 2**

2. Supplementary Tables 1-9 **page 3**

3. Supplementary Methods **page 19**

3.1 Background: The Covid-19 pandemic in the Czech Republic page 19
3.2 Survey of Czech medical doctors: Additional information page 22
3.3 Main experiment: CONSENSUS treatment wording page 24
3.4 Verification of vaccination status: Methods and results page 28
3.5 Regression specification page 32
3.6 Definitions of variables page 34

4. Populated Pre-Analysis Plan **page 36**

1 Supplementary Figure

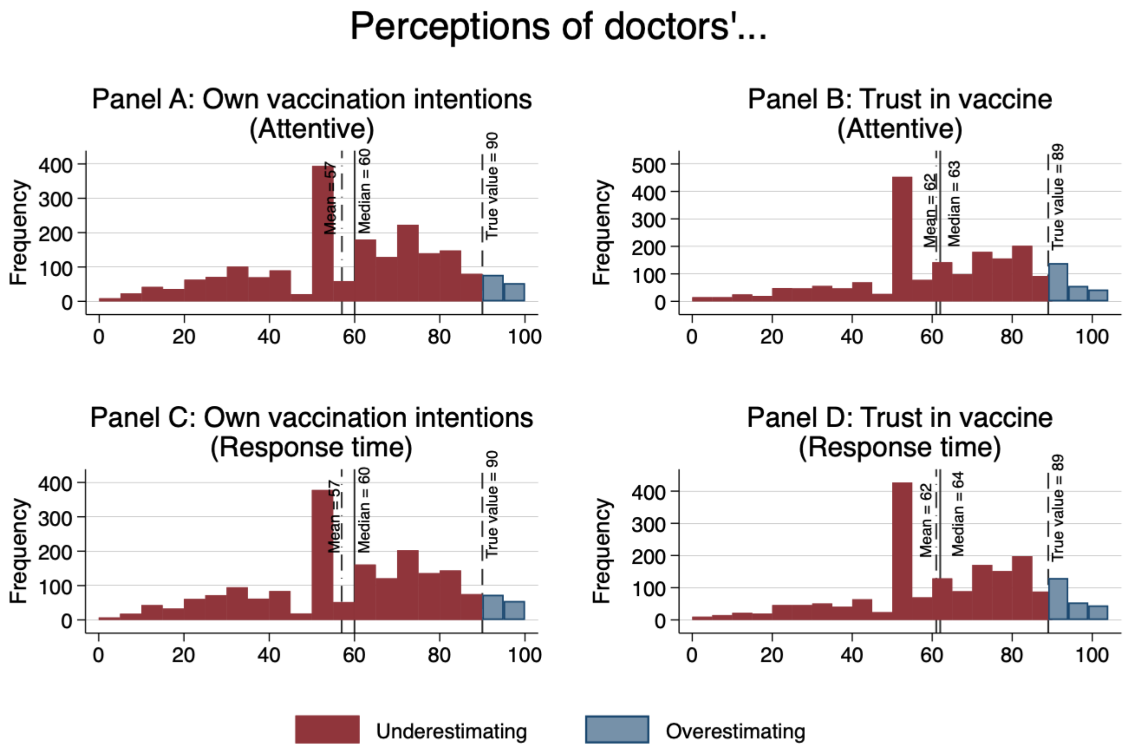

Supplementary Figure 1. Perceptions of views of doctors about the Covid-19 vaccines, excluding inattentive participants (Main Experiment). In **a** and **c**, we report the distributions of respondents' beliefs about what percentage of doctors would like to get vaccinated. In **b** and **d**, we report the distributions of respondents' beliefs about what percentage of doctors trust the approved Covid-19 vaccines. In **a** and **b**, we use data for respondents who passed all attention checks (n=2,009), in **c** and **d**, we exclude data for respondents in the first decile of response time fixed at Wave0 (n=1,892). The dashed line shows the true value, based on the responses of doctors in the Supplementary study. The red (blue) color illuminates the fraction of those who underestimate (overestimate) doctors' own vaccination intentions (in **a** and **c**) and trust in the Covid-19 vaccines (in **b** and **d**).

2 Supplementary Tables

Supplementary Table 1. Comparison of characteristics of the doctors in the Czech Medical Chamber survey (Supplementary Survey) and of all doctors in the Czech Republic.

	(1) Sample mean	(2) SD	(3) Czech Medical Chamber	(4) UZIS administrative data	(5) Difference (3)-(1) (p-value)	(6) Difference (4)-(1) (p-value)
**Female**	0.636	0.481	0.598	0.584	-0.038 (<0.001)	-0.052 (<0.001)
**Age category**						
age cat 18-24	0.001	0.023	0.001	0.001	0.000 (0.038)	0.000 (0.038)
age cat 25-34	0.144	0.352	0.193	0.229	0.049 (<0.001)	0.085 (<0.001)
age cat 35-44	0.200	0.400	0.187	0.217	-0.013 (0.002)	0.017 (<0.001)
age cat 45-54	0.233	0.422	0.183	0.199	-0.050 (<0.001)	-0.034 (<0.001)
age cat 55-64	0.239	0.426	0.169	0.172	-0.070 (<0.001)	-0.067 (<0.001)
age cat 65+	0.184	0.388	0.267	0.182	0.083 (<0.001)	-0.002 (0.587)
**Sector**						
State	0.357	0.479		0.578		0.221 (<0.001)
Private	0.643	0.479		0.596		-0.047 (<0.001)
**Seniority**						
1-10 years	0.196	0.397		0.254		0.058 (<0.001)
11-20 years	0.182	0.385		0.190		0.008 (0.031)
20+ years	0.622	0.485		0.516		-0.106 (<0.001)
**Town size**						
Below 999	0.009	0.093		0.010		0.001 (0.171)
1,000-1,999	0.021	0.145		0.022		0.001 (0.710)
2,000-4,999	0.047	0.212		0.047		0.000 (0.906)
5,000-19,999	0.179	0.383		0.234		0.055 (<0.001)
20,000-99,999	0.317	0.465		0.427		0.110 (<0.001)
Above 100,000	0.427	0.495		0.229		-0.198 (<0.001)
**Region**						
Prague	0.227	0.419	0.230	0.241	0.003 (0.489)	0.014 (0.001)
Central Bohemia	0.087	0.282	0.081	0.100	-0.006 (0.035)	0.013 (<0.001)
South Bohemia	0.044	0.205	0.053	0.056	0.009 (<0.001)	0.012 (<0.001)
Plzeň	0.046	0.210	0.058	0.059	0.012 (<0.001)	0.013 (<0.001)
Karlovy Vary	0.025	0.155	0.022	0.025	-0.003 (0.092)	0.000 (0.831)
Ústí	0.048	0.214	0.056	0.061	0.008 (<0.001)	0.013 (<0.001)
Liberec	0.038	0.192	0.032	0.036	-0.006 (0.002)	-0.002 (0.274)
Hradec Králové	0.052	0.221	0.054	0.055	0.002 (0.310)	0.003 (0.145)
Pardubice	0.043	0.204	0.037	0.044	-0.006 (0.002)	0.001 (0.780)
Vysočina	0.048	0.214	0.037	0.041	-0.011 (<0.001)	-0.007 (0.001)
South Moravia	0.138	0.345	0.130	0.130	-0.008 (0.029)	-0.008 (0.028)
Olomouc	0.091	0.287	0.065	0.070	-0.026 (<0.001)	-0.021 (<0.001)

Zlín	0.041	0.197	0.044	0.050	0.003 (0.083)	0.009 (<0.001)
Moravia-Silesia	0.073	0.260	0.101	0.114	0.028 (<0.001)	0.041 (<0.001)
**Observations**	9,650		57,386	51,638		

Notes: Sample means in column 1. Standard deviations in column 2. Column 3 reports means for the entire population of Czech medical doctors from the database of the Czech Medical Chamber. Column 4 reports means for the entire population of Czech medical doctors from the database of the Institute of Health Information and Statistics (UZIS). Column 5 presents a difference between columns 3 and 1 and a t-test p-value comparing the sample mean to the population mean (CMC). Column 6 presents a difference between columns 4 and 1 and a t-test p-value comparing the sample mean to the population mean (UZIS).

Supplementary Table 2. Share of positive responses of Czech medical doctors to three questions on trust in vaccines, own intentions to get vaccinated, and willingness to recommend the vaccines to their healthy patients (Supplementary Survey among the members of the Czech Medical Chamber).

	(1)	(2)	(3)	(4)
		Sample means		
	Intention	Trust	Recommend	Observations
**Male**	0.907	0.899	0.954	3,511
**Female**	0.894	0.885	0.961	6,139
**Age category**				
age cat 18-24	1.000	0.800	1.000	5
age cat 25-34	0.929	0.912	0.972	1,394
age cat 35-44	0.886	0.881	0.949	1,928
age cat 45-54	0.880	0.871	0.948	2,244
age cat 55-64	0.891	0.886	0.964	2,302
age cat 65+	0.924	0.913	0.965	1,777
**Sector**				
State	0.916	0.901	0.958	3,445
Private	0.890	0.884	0.959	6,205
**Seniority**				
1-10 years	0.916	0.898	0.965	1,894
11-20 years	0.885	0.880	0.949	1,752
20+ years	0.898	0.891	0.959	6,004
**Town size**				
Below 999	0.869	0.893	0.976	84
1,000-1,999	0.879	0.865	0.957	207
2,000-4,999	0.888	0.901	0.965	456
5,000-19,999	0.891	0.878	0.957	1,726
20,000-99,999	0.898	0.887	0.956	3,057
Above 100,000	0.906	0.897	0.960	4,120
**Region**				
Prague	0.913	0.900	0.959	2,191
Central Bohemia	0.920	0.907	0.967	840
South Bohemia	0.900	0.893	0.955	422
Plzeň	0.897	0.886	0.966	447
Karlovy Vary	0.857	0.840	0.945	238
Ústí	0.890	0.886	0.963	464
Liberec	0.913	0.897	0.965	368
Hradec Králové	0.920	0.886	0.950	499
Pardubice	0.876	0.885	0.947	419
Vysočina	0.908	0.905	0.959	465
South Moravia	0.893	0.896	0.959	1,329

Olomouc	0.866	0.866	0.953	876
Zlín	0.903	0.880	0.957	391
Moravia-Silesia	0.884	0.876	0.960	701

Notes: Means in columns 1 to 3. Column 1 reports the share responding "Yes" or "I'm already vaccinated" to the question "Will you personally be interested in getting vaccinated, voluntarily and free of charge, with an approved vaccine against Covid-19?". Column 2 reports the share responding "Yes" to "Do you trust Covid-19 vaccines that have been approved by the European Medicines Agency (EMA) approval process?". Column 3 reports the share responding, "I will actively recommend vaccinations to them, even if they do not ask for my recommendation." or "I will recommend vaccinations to them, if they ask for my recommendation." to the question "Will you recommend Covid-19 vaccination to your healthy patients to whom you would recommend other commonly-used vaccines?" We also estimated weighted means of the same variables, using analytic weights calculated separately for each group of characteristics (in bold) using the population data from UZIS (Column 4 of Supplementary Table 1). All estimates rounded to three digits are identical to those reported in the table.

Supplementary Table 3. Demographic characteristics: summary statistics and randomization check for the fixed sample (Main Experiment, Sample of adult Czech population).

	(1)<br>Fixed sample	(2)<br>CONTROL	(3)<br>CONSENSUS	(4)<br>P-value
**Female**	0.493	0.493	0.493	0.995
**Age category**				
age cat 18-24	0.026	0.03	0.021	0.325
age cat 25-34	0.09	0.089	0.091	0.875
age cat 35-44	0.134	0.139	0.13	0.664
age cat 45-54	0.179	0.176	0.182	0.757
age cat 55-64	0.21	0.216	0.204	0.604
age cat 65+	0.361	0.351	0.371	0.465
**Household size**	2.224	2.182	2.264	0.177
**Number of children**	0.38	0.375	0.384	0.482
**Children missing**	0.034	0.037	0.031	0.574
**Region**				
Prague	0.306	0.323	0.29	0.215
Central Bohemia	0.087	0.072	0.103	0.059
South Bohemia	0.037	0.038	0.036	0.809
Plzeň	0.041	0.042	0.041	0.924
Karlovy Vary	0.02	0.022	0.018	0.633
Ústí	0.066	0.067	0.065	0.903
Liberec	0.044	0.043	0.044	0.966
Hradec Králové	0.04	0.038	0.042	0.731
Pardubice	0.038	0.038	0.037	0.927
Vysočina	0.033	0.025	0.041	0.128
South Moravia	0.092	0.097	0.088	0.587
Olomouc	0.052	0.043	0.06	0.188
Zlín	0.036	0.04	0.033	0.482
Moravia-Silesia	0.106	0.11	0.103	0.661
**Town size**				
Below 999	0.061	0.043	0.078	0.012
1,000-1,999	0.029	0.023	0.034	0.262
2,000-4,999	0.05	0.052	0.049	0.812
5,000-19,999	0.106	0.107	0.106	0.948
20,000-49,999	0.073	0.06	0.085	0.100
50,000-99,999	0.18	0.187	0.173	0.507
Above 100,000	0.501	0.527	0.476	0.075
**Education**				
primary	0.033	0.028	0.037	0.379
lower secondary	0.257	0.244	0.269	0.327
upper secondary	0.379	0.403	0.355	0.085

university	0.332	0.324	0.339	0.596
**Economic status**				
Employee	0.453	0.453	0.453	0.989
Entrepreneur	0.046	0.042	0.05	0.472
Student	0.023	0.025	0.021	0.650
Parental leave	0.029	0.027	0.031	0.663
Retired	0.406	0.406	0.406	0.977
Unemployed	0.03	0.028	0.031	0.796
Other	0.013	0.018	0.008	0.118
**Household income**				
Up to 10,000 CZK	0.008	0.01	0.007	0.498
10,001 - 15,000 CZK	0.077	0.08	0.073	0.648
15,001 - 20,000 CZK	0.095	0.08	0.109	0.087
20,001 - 25,000 CZK	0.074	0.079	0.07	0.570
25,001 - 30,000 CZK	0.104	0.104	0.104	0.975
30,001 - 35,000 CZK	0.13	0.13	0.13	0.994
35,001 - 40,000 CZK	0.116	0.129	0.103	0.154
40,001 - 50,000 CZK	0.114	0.125	0.103	0.211
50,001 - 60,000 CZK	0.092	0.072	0.112	0.015
Over 60,000 CZK	0.079	0.065	0.093	0.075
I don't know / Don't want to say	0.111	0.125	0.096	0.104
**Vaccine intention (Wave -1)**	0.675	0.679	0.671	0.769
**Vaccinated**	0.087	0.097	0.078	0.246
**Beliefs about doctors'**				
Intentions to get vaccinated	57.135	58.564	55.744	0.044
Trust in Covid-19 vaccines	63.114	64.022	62.23	0.102
**Observations**	1,212	598	614	
**Omnibus randomization test of joint significance for all variables above**				
P-value				0.313

Notes: Same as Extended Data Table 1 but for the Fixed sample of participants responding to all Waves 0-11.

Supplementary Table 4. Predictors of beliefs about doctors' intentions of getting a Covid-19 vaccine and about doctors' trust in the Covid-19 vaccine (Main Experiment, Sample of adult Czech population).

	(1)	(2)
**Dependent variable**	Beliefs about doctors' […] Covid-19 vaccine	
	[intentions of getting a]	[trust in]
**Female**	0.855	0.443
	(0.997)	(0.921)
**Age category**		
age cat 25-34	-2.328	2.404
	(3.711)	(3.767)
age cat 35-44	-3.205	2.088
	(3.843)	(3.851)
age cat 45-54	-3.039	0.410
	(3.802)	(3.842)
age cat 55-64	-4.560	-0.240
	(3.918)	(3.895)
age cat 65+	-4.511	0.337
	(4.140)	(4.095)
**Household size**	-0.974	-1.165*
	(0.711)	(0.614)
**Number of children**	1.045	0.470
	(0.905)	(0.796)
**Children missing**	2.890	-1.863
	(2.050)	(1.948)
**Region**		
Central Bohemia	0.482	-0.387
	(2.333)	(2.090)
South Bohemia	-0.029	-3.919
	(2.642)	(2.596)
Plzeň	-0.379	-3.087
	(2.355)	(2.355)
Karlovy Vary	5.177	3.617
	(3.640)	(3.347)
Ústí	-3.653	-3.032
	(2.489)	(2.317)
Liberec	-0.754	-4.357*
	(2.721)	(2.484)
Hradec Králové	-3.980	-3.518
	(2.854)	(2.634)

[Table continues on the next page]

Dependent variable	(1)	(2)
	Beliefs about doctors' […] Covid-19 vaccine	
	[intentions of getting a]	[trust in]
[Table continues here]		
Pardubice	-0.717	-0.574
	(2.681)	(2.531)
Vysočina	-3.567	-4.288
	(3.052)	(2.614)
South Moravia	-2.054	-2.747
	(1.873)	(1.738)
Olomouc	-1.389	1.051
	(2.670)	(2.271)
Zlín	-1.682	-4.971*
	(2.750)	(2.671)
Moravia-Silesia	-2.162	-3.088*
	(1.834)	(1.634)
**Town size**		
1,000-1,999	-1.589	-0.500
	(3.081)	(2.839)
2,000-4,999	0.076	-0.213
	(2.650)	(2.332)
5,000-19,999	-1.518	-1.365
	(2.223)	(2.114)
20,000-49,999	-1.643	-0.183
	(2.559)	(2.368)
50,000-99,999	-1.459	-1.080
	(2.223)	(2.067)
Above 100,000	1.190	0.608
	(2.369)	(2.110)
**Education**		
lower secondary	-3.088	-0.935
	(2.410)	(2.420)
upper secondary	-2.739	1.888
	(2.356)	(2.335)
university	-2.013	3.766
	(2.458)	(2.394)

[Table continues on the next page]

	(1)	(2)
**Dependent variable**	Beliefs about doctors' […] Covid-19 vaccine	
	[intentions of getting a]	[trust in]
**[Table continues here]**		
**Economic status**		
Entrepreneur	4.385*	3.191
	(2.272)	(2.055)
Student	1.149	5.375
	(3.894)	(4.108)
Parental leave	-1.530	-2.402
	(2.563)	(2.283)
Retired	3.472**	4.344***
	(1.742)	(1.643)
Unemployed	-1.526	-1.614
	(2.827)	(2.646)
Other	-0.869	5.376
	(3.022)	(3.475)
**Household income**		
10,001 - 15,000 CZK	-2.108	1.522
	(5.469)	(4.333)
15,001 - 20,000 CZK	-1.455	0.891
	(5.356)	(4.093)
20,001 - 25,000 CZK	2.474	1.788
	(5.466)	(4.264)
25,001 - 30,000 CZK	3.061	5.214
	(5.348)	(4.102)
30,001 - 35,000 CZK	4.825	5.407
	(5.364)	(4.080)
35,001 - 40,000 CZK	4.450	8.227**
	(5.383)	(4.103)
40,001 - 50,000 CZK	3.929	8.783**
	(5.431)	(4.123)
50,001 - 60,000 CZK	6.049	9.984**
	(5.519)	(4.192)
Over 60,000 CZK	7.862	11.219***
	(5.564)	(4.275)
I don't know / Don't want to say	2.684	3.847
	(5.387)	(4.138)

[Table continues on the next page]

	(1)	(2)
**Dependent variable**	Beliefs about doctors' […] Covid-19 vaccine	
	[intentions of getting a]	[trust in]
**[Table continues here]**		
**Vaccine intention (Wave -1)**	10.626***	16.648***
	(1.074)	(1.018)
**Vaccine intention (Wave -1) missing**	0.962	9.438**
	(4.730)	(4.244)
**Vaccinated**	3.912**	4.872***
	(1.698)	(1.354)
Constant	53.719***	44.640***
	(7.099)	(6.013)
Observations	2,101	2,101
R-squared	0.105	0.243

Notes: OLS coefficients. Huber-White robust standard errors in parentheses. The dependent variable in Column 1 is the respondent's elicited belief about the percentage of doctors who would like to get vaccinated. The dependent variable in Column 2 is the respondent's elicited belief about the percentage of doctors who trust the approved Covid-19 vaccines. Both dependent variables range between 0 and 100. Wave0 full sample used. In both columns, the set of controls is the same as in Figure 3 except for the Wave0 belief measures, which are excluded. T-test p-values (two-sided) reported as *p<0.10; **p<0.05; ***p<0.01. No adjustments for multiple comparisons.

Supplementary Table 5. Effect of the CONSENSUS condition on respondents' beliefs about doctors' intentions of getting a Covid-19 vaccine and about doctors' trust in the Covid-19 vaccine.

	(1)	(2)	(3)	(4)	(5)	(6)	(7)
Sample	Full	Fixed	Attentive	Underestimating	Overestimating	Prior intention: Not to get vaccinated	Prior intention: To get vaccinated
**Panel A: Dependent variable Beliefs about doctors' trust in Covid-19 vaccines**							
**Linear probability model with pre-registered set of controls**							
CONSENSUS	5.160***	4.768***	5.185***	5.840***	-1.707	7.572***	3.655***
	(0.862)	(1.048)	(0.871)	(0.915)	(1.613)	(1.741)	(0.958)
Observations	1,940	1,212	1,901	1,714	226	673	1,267
CONTROL mean	66.643	67.834	66.626	63.676	88.704	53.601	73.492
R-squared	0.233	0.250	0.236	0.218	0.292	0.132	0.080
Comparison chi-sq (p-value)				20.06 (0.000)		4.16 (0.041)	
**Double-selection LASSO linear regression**							
CONSENSUS	6.099***	5.407***	6.004***	6.955***	-1.043	8.464***	4.895***
	(0.710)	(0.857)	(0.716)	(0.775)	(1.536)	(1.417)	(0.776)
Comparison chi-sq (p-value)				21.83 (0.000)		4.90 (0.027)	
**Panel B: Dependent variable Beliefs about doctors' intentions to get vaccinated against Covid-19**							
**Linear probability model with pre-registered set of controls**							
CONSENSUS	6.461***	6.404***	6.432***	6.926***	0.559	7.801***	5.689***
	(0.921)	(1.159)	(0.930)	(0.947)	(2.996)	(1.706)	(1.103)
Observations	1,940	1,212	1,901	1,814	126	673	1,267
CONTROL mean	62.980	63.555	63.022	61.378	74.889	54.740	67.307
R-squared	0.138	0.142	0.141	0.148	0.415	0.117	0.091
Comparison chi-sq (p-value)				6.44 (0.011)		1.15 (0.283)	
**Double-selection LASSO linear regression**							
CONSENSUS	7.465***	7.247***	7.353***	7.324***	6.545**	8.480***	6.833***
	(0.824)	(1.029)	(0.830)	(0.881)	(2.618)	(1.447)	(1.008)
Comparison chi-sq (p-value)				6.72 (0.010)		0.88 (0.349)	

Notes: OLS coefficients. Huber-White robust standard errors in parentheses. The dependent variable in all columns of Panel A is the respondent's guess about the percentage of doctors who would like to get vaccinated. The dependent variable in all columns of Panel B is the respondent's guess about the percentage of doctors' who trust the approved Covid-19 vaccines. Both dependent variables range between 0 and 100 and were measured in Wave1. Column 1 uses the full sample. Column 2 uses a sample of respondents participating in all 11 waves. Column 3 restricts the full sample to respondents who passed all attention checks embedded in the survey. Columns 4 and 5 restrict the sample to respondents who underestimate or overestimate trust in the Covid-19 vaccines (Panel A) and doctors' intentions to get vaccinated (Panel B). Columns 6 and 7 restrict the sample to respondents without and with intentions to get vaccinated prior to Wave0, respectively. In all columns we use the pre-registered set of controls. Estimated coefficients from a double-selection LASSO linear regression (dsregress command in Stata 17), selected from a set of covariates in Extended Data Table 1, are reported at the bottom of each panel. Rows titled "Comparison" in each panel report a chi-square statistic and a p-value for a test of equivalence of coefficients across two respective models estimated using seemingly unrelated regressions (suest command in Stata 17). For LASSO selected controls, we use OLS models with controls selected by LASSO. T-test p-values (two-sided) reported as *p<0.10; **p<0.05; ***p<0.01. No adjustments for multiple comparisons.

Supplementary Table 6. Effect of the CONSENSUS condition on respondents' vaccination intentions.

	(1)	(2)	(3)
Dependent variable		Vaccination intentions	
Sample	Full	Fixed	Attentive
**Panel A: Wave 0**			
**Linear probability model with pre-registered set of controls**			
CONSENSUS	0.026**	0.053***	0.029**
	(0.013)	(0.016)	(0.013)
Observations	2,101	1,212	2,009
CONTROL mean	0.642	0.659	0.646
R-squared	0.640	0.650	0.655
**Double-selection LASSO linear regression**			
	0.030**	0.057***	0.031**
	(0.013)	(0.016)	(0.013)
**Panel B: Wave 1**			
**Linear probability model with pre-registered set of controls**			
CONSENSUS	0.024*	0.048***	0.027**
	(0.013)	(0.017)	(0.013)
Observations	1,940	1,212	1,901
CONTROL mean	0.684	0.691	0.682
R-squared	0.615	0.618	0.619
**Double-selection LASSO linear regression**			
	0.028**	0.051***	0.028**
	(0.013)	(0.016)	(0.013)

Notes: OLS coefficients. Huber-White robust standard errors in parentheses. The dependent variable in all columns is an indicator for vaccination intentions, equal to 1 if the respondent reported already being vaccinated or registered for the vaccine, or being willing to get vaccinated. Column 1 uses the full sample. Column 2 uses a sample of respondents participating in all 11 waves. Column 3 restricts the full sample to respondents who passed all attention checks embedded in the survey. Panel A reports results for Wave0 responses. Panel B reports results for Wave1 responses. In all columns we use the pre-registered set of controls. Estimated coefficients from a double-selection LASSO linear regression (dsregress command in Stata 17) selecting from a set of covariates in Extended Data Table 1 are reported in the bottom parts of each panel. T-test p-values (two-sided) reported as *p<0.10; **p<0.05; ***p<0.01. No adjustments for multiple comparisons.

Supplementary Table 7. Effect of the CONSENSUS condition on respondents' vaccination take-up: difference-in-differences estimation.

	(1)	(2)	(3)	(4)	(5)	(6)
Dependent variable	Vaccinated					
Sample	Full	Full	Full	Fixed	Fixed	Fixed
**Waves 0, and 6-11**						
CONSENSUS x Wave 6-11	0.056***	0.054***	0.055***	0.069***	0.070***	0.070***
	(0.021)	(0.021)	(0.021)	(0.025)	(0.023)	(0.023)
Wave 6-11	0.648***	0.639***	0.639***	0.651***	0.629***	0.631***
	(0.015)	(0.015)	(0.015)	(0.018)	(0.017)	(0.017)
CONSENSUS	-0.019	-0.020	-0.009	-0.019	-0.019	-0.008
	(0.012)	(0.014)	(0.011)	(0.012)	(0.014)	(0.011)
Controls	No	Pre-registered	LASSO	No	Pre-registered	LASSO
Observations	12,383	12,383	12,383	9,373	9,373	9,373
R-squared	0.282	0.482		0.347	0.533	

Notes: OLS coefficients. Standard errors clustered at the respondent level in parentheses. The dependent variable in all columns is an indicator for vaccination take-up, equal to 1 if the respondent reported having obtained at least one dose of a vaccine against Covid-19. Wave 6-11 is an indicator equal to 1 if the observation is from Wave 6 - 11. CONSENSUS x Wave 6-11 is an interaction term of interest in the difference in differences specification. Columns 1-3 use the full sample. Columns 4-6 use a sample of respondents participating in all 11 waves. We use data on take up from Waves 6-11 when vaccines were available for all adults and the baseline data on take up is from Wave 0. Columns 1 and 4 use no additional controls. Columns 2 and 5 use the pre-registered set of controls. Columns 3 and 6 report coefficients from a double-selection LASSO linear regression (dsregress command in Stata 17) selecting from a set of covariates in Extended Data Table 1. T-test p-values (two-sided) reported as *p<0.10; **p<0.05; ***p<0.01. No adjustments for multiple comparisons.

Supplementary Table 8. Respondent attrition by round.

	(1)	(2)	(3)	(4)	(5)	(6)	(7)	(8)	(9)	(10)	(11)	(12)
Dependent variable	Participation in wave…											
	1	2	3	4	5	6	7	8	9	10	11	1-11
CONSENSUS	0.001	-0.006	0.003	0.010	0.007	-0.005	0.004	-0.010	0.005	0.011	0.007	0.016
	(0.012)	(0.012)	(0.013)	(0.014)	(0.015)	(0.018)	(0.016)	(0.016)	(0.017)	(0.018)	(0.015)	(0.022)
Observations	2,101	2,101	2,101	2,101	2,101	2,101	2,101	2,101	2,101	2,101	2,101	2,101
CONTROL mean	0.923	0.926	0.901	0.880	0.850	0.774	0.841	0.835	0.823	0.761	0.853	0.569
R-squared	0.000	0.000	0.000	0.000	0.000	0.000	0.000	0.000	0.000	0.000	0.000	0.000
**Omnibus test for a joint effect of interaction terms of CONSENSUS with pre-specified set of controls**												
P-value	0.629	0.958	0.332	0.734	0.521	0.804	0.159	0.326	0.113	0.174	0.949	0.869

Notes: OLS coefficients. Huber-White robust standard errors in parentheses. The dependent variable is an indicator for whether a respondent participated in a respective wave (Wave1 in Column 1 to Wave11 in Column 11, participation in all waves in Column 12). The omnibus randomization test of joint significance presents a p-value of an F-test (two-sided) for joint significance of a sum of coefficients for the CONSENSUS condition and of all interactions of pre-specified controls with CONSENSUS in an OLS regression, with participation in a respective wave as a dependent variable and CONSENSUS, pre-specified set of controls, and interaction terms of CONSENSUS and pre-specified set of controls as independent variables. No adjustments for multiple comparisons.

Supplementary Table 9. Effects of the CONSENSUS condition on take-up: More detailed analysis, based on whether vaccination status verified (ordered and multinomial logit).

	(1)	(2)	(3)	(4)	(5)	(6)
Specification		Ordered logit			Multinomial logit	
Verification	Third party verification	Certificate verification	Third party OR certificate verification	Third party verification	Certificate verification	Third party OR certificate verification
Dependent variable				Vaccinated		
**Waves 6-11, Effects of CONSENSUS on the prevalence of the following categories**						
Vaccinated, verified	0.048***	0.034**	0.038**	0.047***	0.030*	0.038**
	(0.017)	(0.017)	(0.016)	(0.017)	(0.018)	(0.016)
	[0.004]	[0.045]	[0.016]	[0.007]	[0.093]	[0.019]
Vaccinated, not verified	-0.005***	-0.005**	-0.002**	-0.009	0.008	-0.000
	(0.002)	(0.002)	(0.001)	(0.010)	(0.012)	(0.007)
	[0.006]	[0.047]	[0.021]	[0.354]	[0.520]	[0.993]
Not vaccinated	-0.043***	-0.030**	-0.036**	-0.037**	-0.038**	-0.038**
	(0.015)	(0.015)	(0.015)	(0.015)	(0.015)	(0.015)
	[0.004]	[0.045]	[0.016]	[0.015]	[0.014]	[0.014]

Notes: Marginal effects for ordered logit (Columns 1-3) and multinomial logit (Columns 4-6) estimates. Delta-method standard errors in parentheses. Z-test (two-sided) p-values in square brackets, *p<0.10; **p<0.05; ***p<0.01. The dependent variable in all columns is a variable for vaccination take-up. The variable equals to 2 if the respondent reported having obtained at least one dose of a vaccine against Covid-19 and the self-report has been verified with either of the verification methods (See Supplementary Information Section 3.4 for more details on verification). It equals to 1 if the respondent reported having obtained at least one dose of a vaccine against Covid-19 but this has not been verified. It equals to 0 if the respondent reported not having obtained any vaccine against Covid-19. Full sample used. In all columns we use the pre-registered set of controls. All columns include wave fixed effects. Standard errors are clustered at an individual level. No adjustments for multiple comparisons.

3 Supplementary Methods

3.1 Background: The Covid-19 pandemic in the Czech Republic

The Czech Republic is a landlocked country in Central Europe, bordering Germany, Austria, Slovakia, and Poland. The population is around 10.7 million. The Czech Republic is a parliamentary democracy and it joined the EU in 2004. The 2018 GDP per capita (PPP) was around USD 40,000 (or 90.6% of the EU average).

The population of the Czech Republic was strongly impacted by the Covid-19 pandemic. Up to December 2021, there were four major waves of the disease, during which 21% of the population has been officially confirmed to have been infected with Covid-19, with many more undetected cases likely. More than 33 thousand people (approximately 0.3% of the total population) have died of Covid-19, resulting in the Czech Republic's ranking among the ten worst countries in the world as measured by deaths per 100,000 population (Johns Hopkins University, Coronavirus Resource Center).

We use data from twelve waves of longitudinal data collection. The information intervention was implemented in Wave 0, which took place in mid-March 2021, shortly after the peak of the third wave of Covid-19. The situation then gradually improved during Waves 2-5 (March-June 2021), and became relatively calm during Waves 6-9 (July-October 2021). The last two Waves 10-11 took place during the fourth wave of Covid-19 (November 2021). Extended Data Figure 3 displays the development of the Covid-19 situation measured by the number of newly confirmed cases per 100,000 population and the timing of the twelve waves of data collection.

Vaccination. The Covid-19 vaccine rollout in the Czech Republic was launched in January 2021. It began with those considered the most vulnerable and later expanded by age and other groups. Specific groups could register in the reservation system on:

- January 15, 2021: persons 80 and older
- January 26, 2021: healthcare professionals
- February 27, 2021: school staff
- March 1, 2021: persons 70 and older
- March 24, 2021: persons with severe chronic diseases
- March 29, 2021: critical infrastructure staff
- April 7, 2021: social services staff
- April 12, 2021: persons with less severe chronic diseases
- April 14, 2021: persons 65 and older
- April 23, 2021: persons 60 and older
- April 28, 2021: persons 55 and older
- May 3, 2021: university academic staff and people caring for a dependent person
- May 5, 2021: persons 50 and older
- May 11, 2021: persons 45 and older
- May 17, 2021: persons 40 and older
- May 24, 2021: persons 35 and older
- May 26, 2021: persons 30 and older
- June 4, 2021: persons 16 and older

- July 1, 2021: persons 12 and older
- December 13, 2021: persons 5 and older

In early stages of vaccination, accessing a vaccine was rather difficult because supplies were limited and demand was high. Registered persons typically had to wait several weeks for a vaccination. The situation gradually improved, and during the summer it became relatively easy to get vaccinated. Supplementary Figure 2 displays a timeline of the numbers of people waiting for their first and second doses.

In our analysis, we report as the main results the estimates of the effect of the CONSENSUS condition on vaccine take-up in Waves 6-11, i.e. in the period July-November 2021. During this period, as described above, the vaccine was easily available for the whole adult population. We do not include Wave 5 into these estimates, which was launched on June 21, 2021, i.e. 17 days after the registration for vaccination was open for all adults. It is unlikely that everyone interested in vaccination could get the first dose of the vaccine since there was still a significant waiting time to obtain the vaccine at that time.

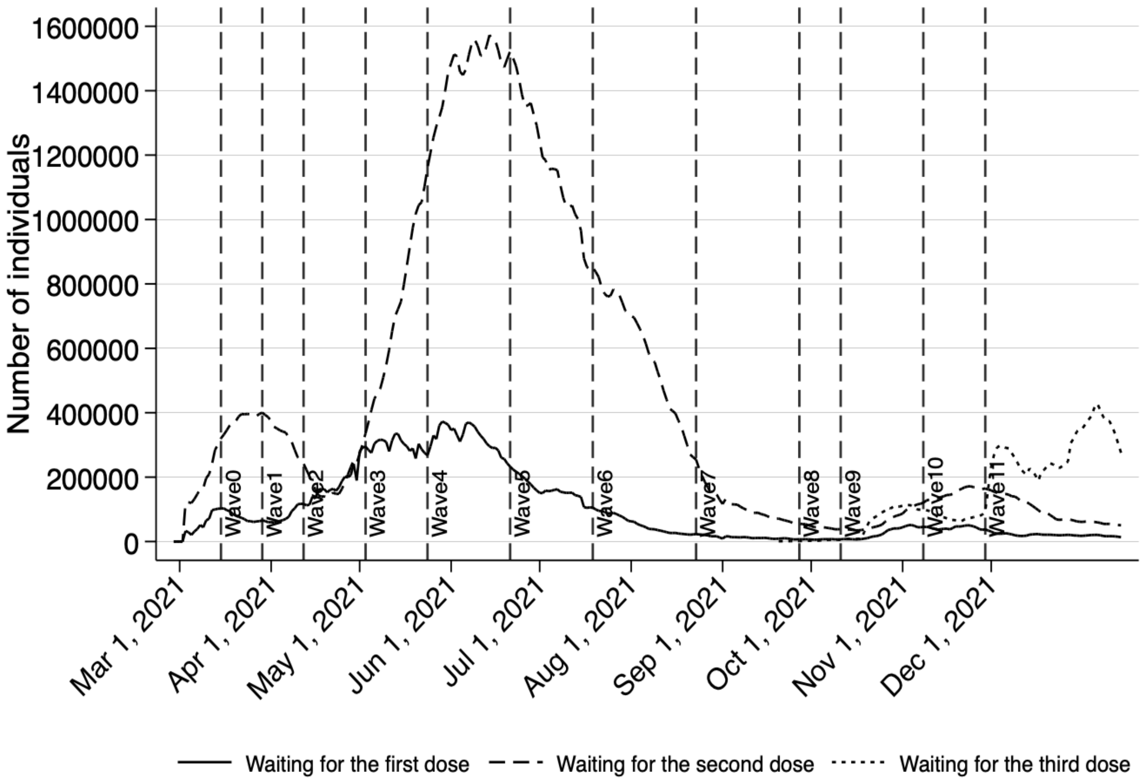

Supplementary Figure 2. Timeline of the numbers of people waiting for their first, second and third doses. The solid, dashed and dotted lines represent the number of Czechs waiting for their first, second and third dose, respectively. Source: open data from the Czech Ministry of Health (https://ockovani.opendatalab.cz/statistiky#queue_graph, Accessed on January 12, 2022)[1] and https://ockovani.opendatalab.cz/, ISSN 2787-9925 - http://aleph.techlib.cz/F/?func=direct&doc_number=000017426&local_base=STK02 (Accessed on January 12, 2022).

As compared to other countries in Europe, at the beginning of December 2021, at 73.8.7%, the adult uptake rate of at least the first dose in the Czech Republic was somewhat lower that the average adult uptake in the 30 countries of the European Economic Area (83.2%). The development of vaccination rates over time has also been somewhat slower in the Czech Republic than in the EEA overall (Supplementary Figure 3).

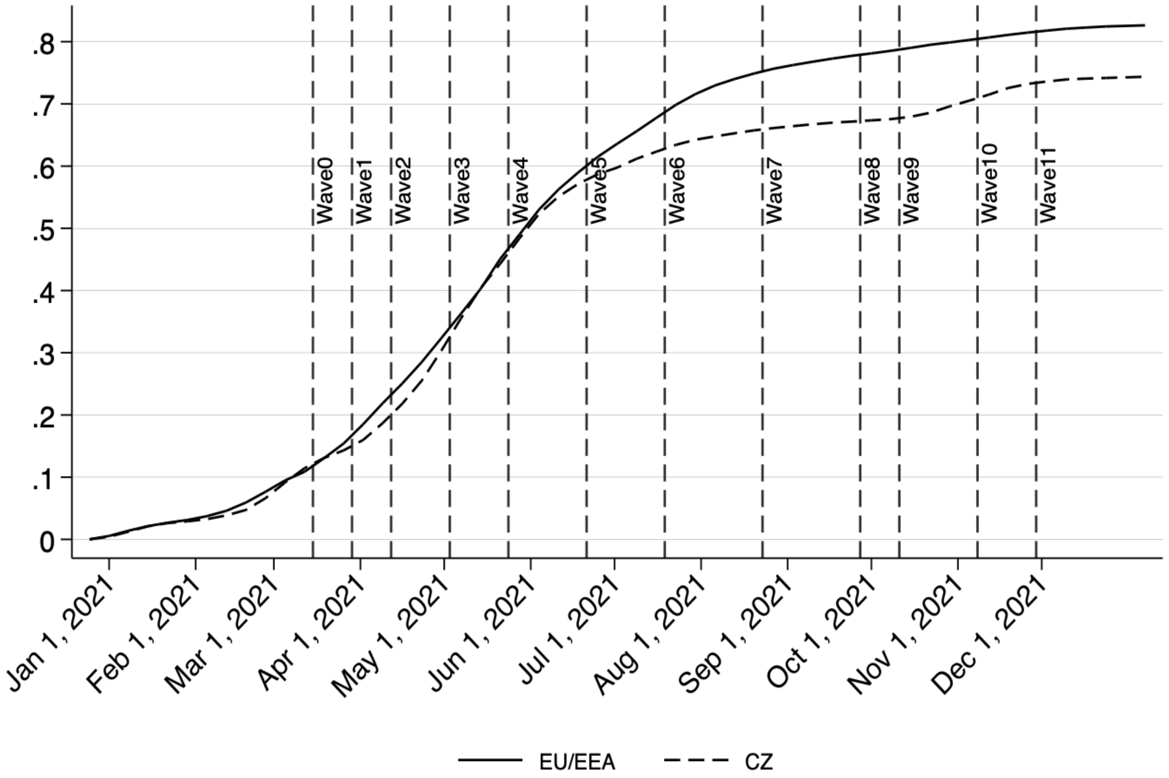

Supplementary Figure 3. Cumulative fraction of adult (18+) EU/EEA and Czech population receiving at least one dose of Covid-19 vaccines by reporting week. Data source: European Centre for Disease Prevention and Control (https://www.ecdc.europa.eu/en/publications-data/data-covid-19-vaccination-eu-eea, Accessed on January 12, 2022).

3.2 Survey among Czech medical doctors: Additional information

Together with the Czech Medical Chamber (CMC), we administered a survey via email to all members of the CMC who communicate with the organization electronically (70%). All medical doctors are obliged to be members of the CMC by the Czech law. Data collection took place between February 11 and 24, 2021. The median duration of the survey was 4 minutes. We used the online Qualtrics platform for data collection.

The survey link was opened by 11,655 respondents. Of these, 1,164 answered that they do not currently work in healthcare, 83 workers in healthcare answered that they are not medical doctors, and 92 answered that they do not work in the Czech Republic. We excluded these respondents from the analysis. 666 respondents did not complete the survey. In the analysis, we work with a sample of 9,650 medical doctors, which is 24% of the doctors surveyed.

The wording of the survey module is as follows (translated from original Czech):

Introduction and informed consent: Hello, we would like to ask you for 4 minutes of your time to fill out a short online survey. The survey focuses on the attitudes of Czech medical doctors on the topic of anti-epidemic measures against the spread of SARS-CoV-2 and Covid-19, specifically on vaccination. The questionnaire was created in cooperation with the “Doctors Help the Czech Republic” initiative, the "Snow" initiative, and the Faculty of Economics at the University of Munich (Ludwig-Maximilians-Universität München). The survey is sponsored by the Czech Medical Chamber (CMC).
All information provided in the survey is completely anonymous. The results will be presented only in aggregate (i.e. it will not be possible to identify you as an individual). We therefore ask for your honest answers. You can close the survey at any time by closing the browser window so that your data is not recorded. By pressing the "Continue" button, you confirm that you have read this text and agree to your participation in the survey. Thank you very much for your time. [Continue]

1. Do you currently work in healthcare? (If you are on maternity or parental leave and have a healthcare contract, answer Yes. [Yes / No]
   Skip To: End of Survey if [No]
2. Gender [Male / Female / Other]
3. What age category do you belong to? [18–24 / 25–34 / 35–44 / 45-54 / 55–64 / 65 or more]
4. Are you a…? [doctor / nurse / brother / another healthcare worker]
5. Do you work mainly in a … health facility? [state / non-state]
6. Medical field [General Practice / Dentistry / Internal Medicine / Other]
7. How many years have you worked in healthcare? [1-5 years / 6-10 years / 11-20 years / More than 20 years]
8. The size of the municipality in which your workplace is located [Less than 999 inhabitants / 1,000–1,999 inhabitants / 2,000-4,999 inhabitants / 5,000–19,999 inhabitants / 20,000-49,999 inhabitants / 50,000-99,999 inhabitants / Over 100,000 inhabitants]
9. The region in which your workplace is located [Prague / Central Bohemian Region / South Bohemian Region / Pilsen / Karlovy Vary / Ústí nad Labem / Liberec / Hradec Králové / Pardubice Region / Vysočina Region / South Moravian Region / Olomouc / Moravskoslezký / Zlínský / I work outside the Czech Republic]

We would now like to ask you a few questions on anti-epidemiological measures against the spread of SARS-CoV-2 and Covid-19, specifically on vaccination.

10. Have you contracted Covid-19? [Yes, multiple times / Yes, once / Ne / I'm not sure]
11. Is application of vaccines a part of your job? [Yes / No]
12. How well do you feel informed about the Covid-19 vaccines that have undergone the European Medicines Agency (EMA) approval process? [I am actively searching for information, or I have searched for it, I have enough information / I am actively searching for information, or I have searched for it, but I do not have enough of it / I am not actively searching for information, nor have I searched for it]
13. Will you personally be interested in getting vaccinated, voluntarily, and free of charge, with an approved vaccine against Covid-19? [Yes / No / I'm not sure / I have already been vaccinated]
14. *Display if response to previous question [No / I'm not sure]:* Why will you not get vaccinated against Covid-19, or why do you hesitate? [*Select multiple*: I am worried about side effects / I am not afraid of coronavirus infection / In general, I do not trust vaccination / I do not believe in meaningfulness if the vaccination is not widespread / I wouldn't want to see a doctor because of that / I trust the ability of the immune system to fight Covid-19 / I am concerned because of the information from the media, social networks, etc. / Other reason]
15. Do you trust Covid-19 vaccines that have been approved by the European Medicines Agency (EMA) approval process? [Yes, at least one of the vaccines / No / I'm not sure.]
16. Display if response to previous question [No / I'm not sure]: Why don't you trust EMA-approved vaccines? [Select multiple: Vaccine development was too fast / The vaccine approval process was too fast / I am worried about side effects / In general, I do not trust vaccination / Covid-19 is not such a serious disease that people need to be vaccinated against it / Covid-19 does not actually exist / I trust the ability of the immune system to fight Covid-19 / I am concerned because of the information from the media, social networks, etc. / Other reason]
17. Will you recommend Covid-19 vaccination to your healthy patients to whom you would recommend other commonly used vaccines? [I will actively recommend it even without being asked / I will recommend it when asked / I will not recommend it when asked / I will actively not recommend it even without being asked]
18. Do you think it is right to vaccinate as many people as possible against Covid-19? [Yes / No / I'm not sure]
19. If response to question "Is application of vaccines a part of your job?" [Yes]: You have indicated that vaccination is part of your job. If a Covid-19 vaccine is available in your practice, will you be actively involved in this vaccination? [Yes / No / I'm not sure]
20. *If response to previous question [No / I'm not sure]:* You stated that: In your practice you are vaccinating and at the same time that you are not convinced whether you would be involved in the vaccination campaign against Covid-19. Can you briefly describe your reasons for this attitude? [Open text]
21. How effective do you think the Pfizer / BioNTech vaccine currently used in the Czech Republic is? (In percent) *[Hint: 0% will not protect anyone who is vaccinated 100% will protect everyone who is vaccinated]*
22. And the last question: What do you think will be the side effects of the Pfizer / BioNTech vaccine? [Milder than commonly used vaccines / Similar to commonly used vaccines / More severe than commonly used vaccines / Much more serious than commonly used vaccines / I'm not sure]

3.3 Main experiment: CONSENSUS treatment wording

The wording of the CONSENSUS treatment is as follows:

Treatment group: 4 slides with text (restriction to minimum of 5 seconds on each page)

Translation to English from the original Czech version

Slide 1: In recent weeks, the Czech Medical Chamber conducted a survey among all medical doctors in the Czech Republic regarding vaccination. Almost 10,000 medical doctors from all parts of the country, from small and large municipalities, and from all age categories responded to the survey. We would like to share the results with you. The results do not differ across different groups of physicians.

Slide 2: The interest of Czech medical doctors in vaccination against Covid-19 is large. 90% of medical doctors are already vaccinated or are interested in getting vaccinated. Only 4% of doctors would not get vaccinated.

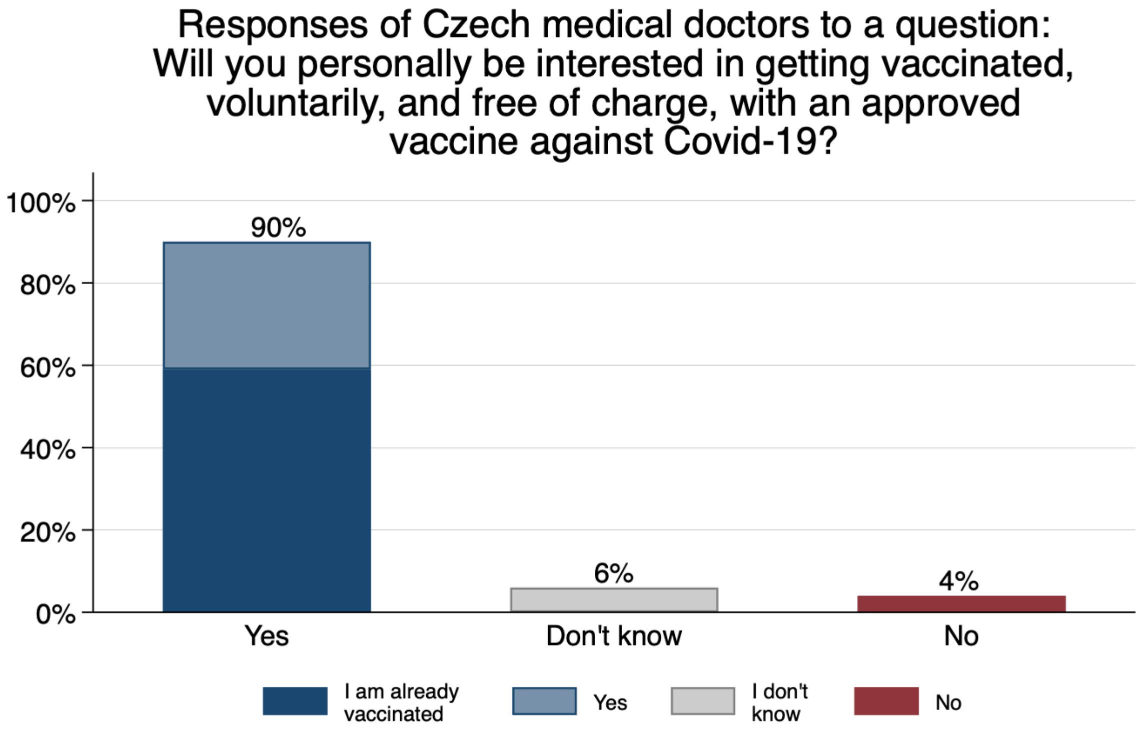

Slide 3: Most Czech medical doctors would recommend vaccination against Covid-19 to their healthy patients. 96% of physicians would recommend vaccination to their healthy patients either on their own initiative or if their patients ask for their opinion.

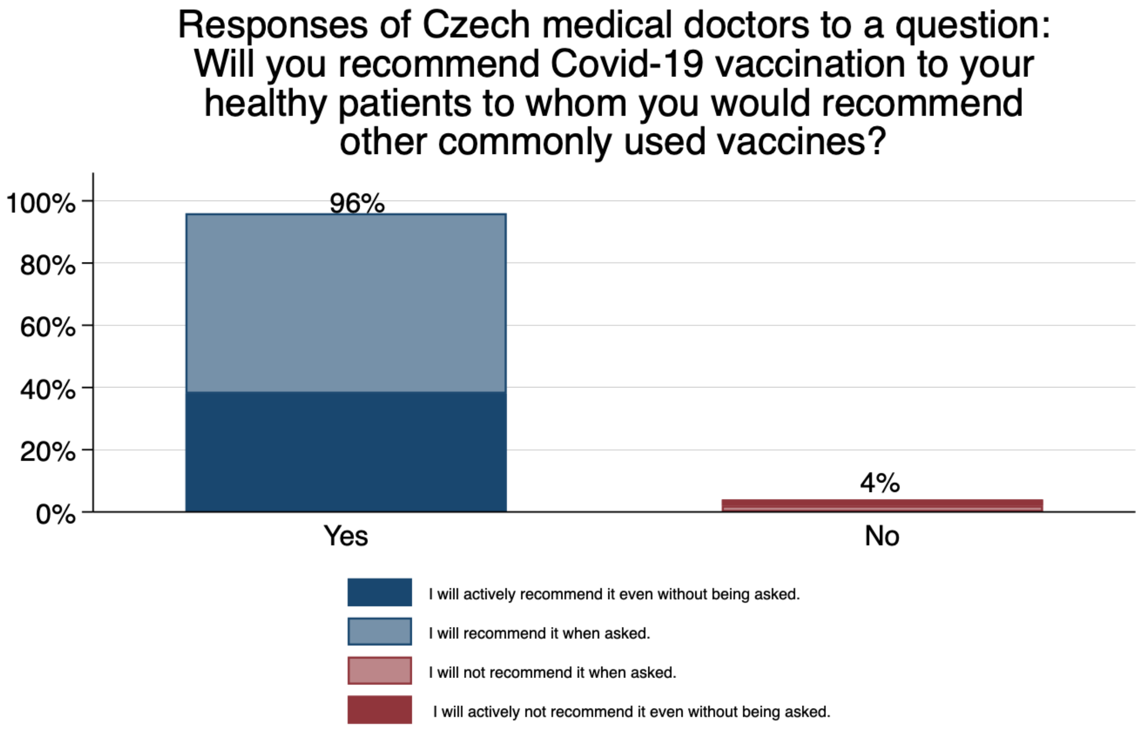

Slide 4: Czech medical doctors' trust in Covid-19 vaccines is strong. 89% of doctors trust vaccines approved by the European Medicines Agency. Only 2% of doctors do not trust them.

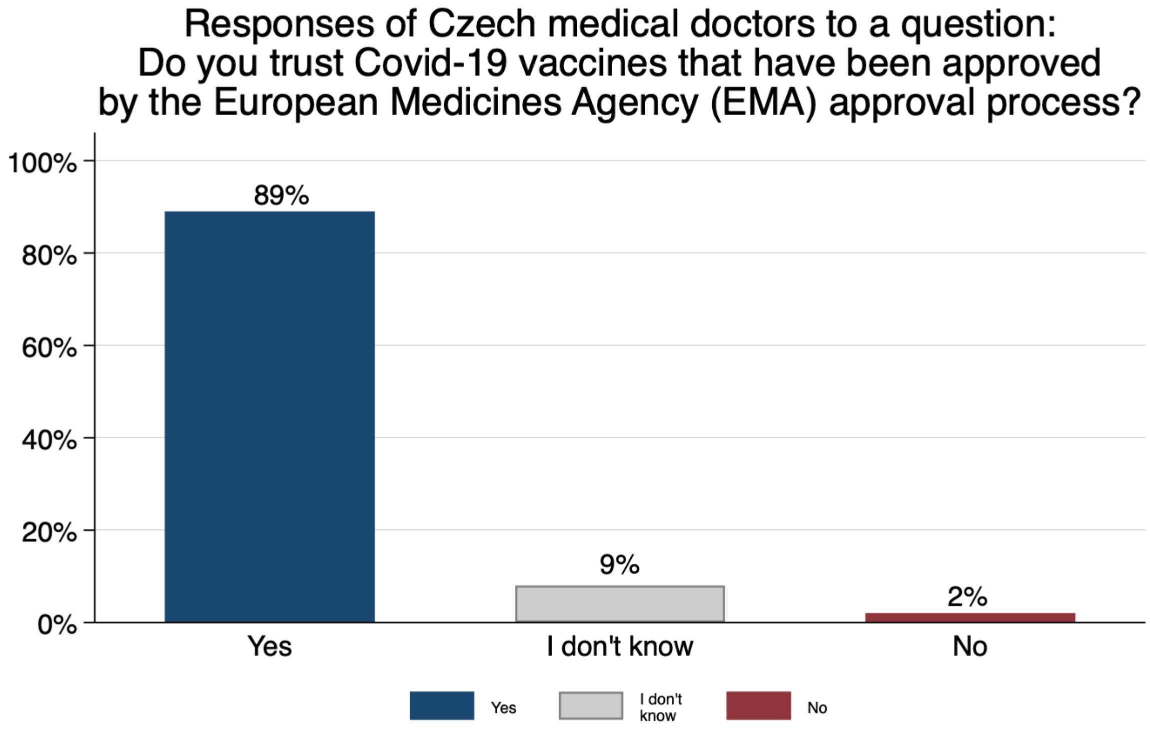

Original Czech version

Slide 1: Česká lékařská komora provedla v minulých týdnech průzkum mezi všemi lékaři a lékařkami v ČR ohledně očkování. Na průzkum odpovědělo téměř 10 tisíc lékařů a lékařek ze všech částí republiky, z malých i velkých obcí, ze všech věkových kategorií. Rádi bychom vás seznámili s výsledky. Výsledky se nijak neliší napříč různými skupinami lékařů.

Slide 2: Zájem českých lékařů o očkování proti nemoci Covid-19 je velký. 90 % lékařů je již očkovaných a nebo má zájem se nechat očkovat. Pouze 4 % lékařů by se očkovat nenechalo.

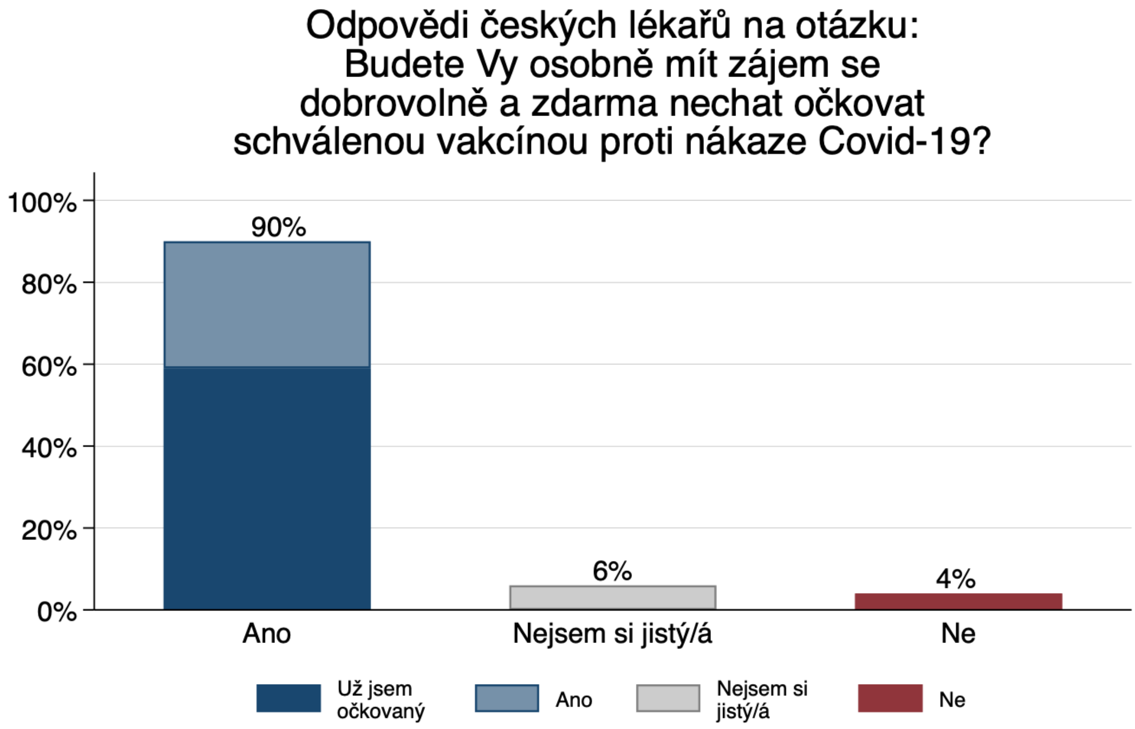

Slide 3: Většina českých lékařů by očkování proti nemoci Covid-19 svým zdravým pacientům doporučila. 96 % lékařů by očkování svým zdravým pacientům doporučila buď z vlastní iniciativy nebo pokud se jich jejich pacienti na názor sami zeptají.

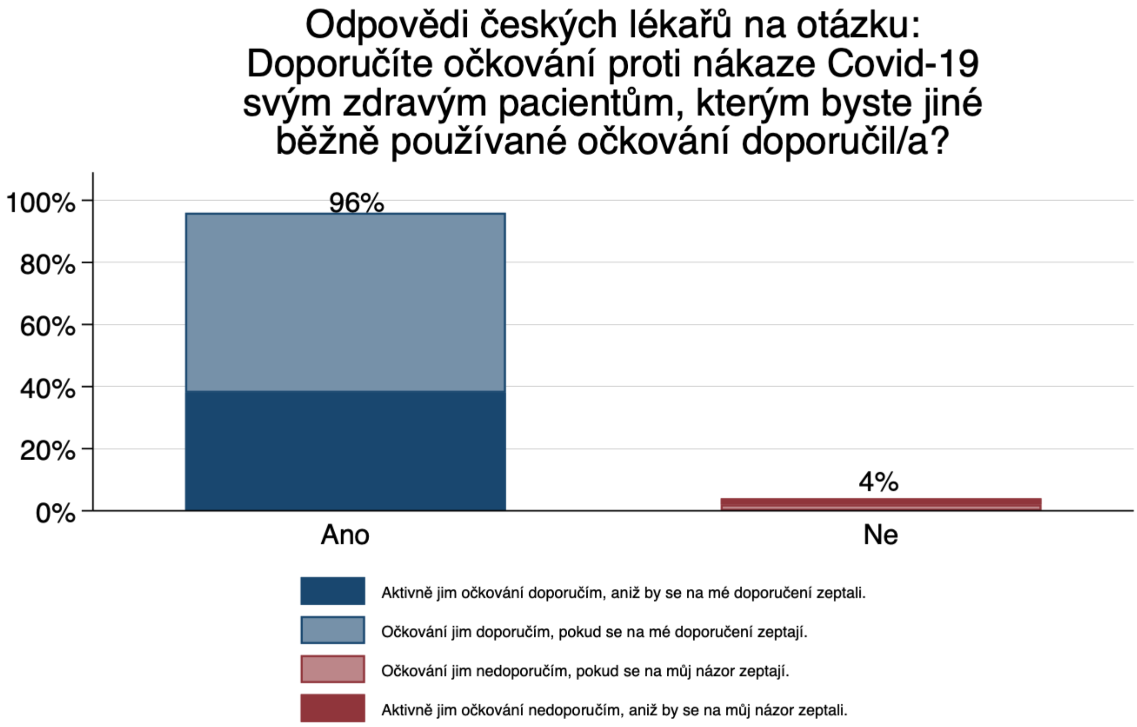

Slide 4: Důvěra českých lékařů ve vakcíny proti nemoci Covid-19 je silná. 89 % lékařů vakcínám schváleným Evropskou agenturou pro léčivé příspěvky důvěřuje. Pouze 2 % lékařů jim nedůvěřují.

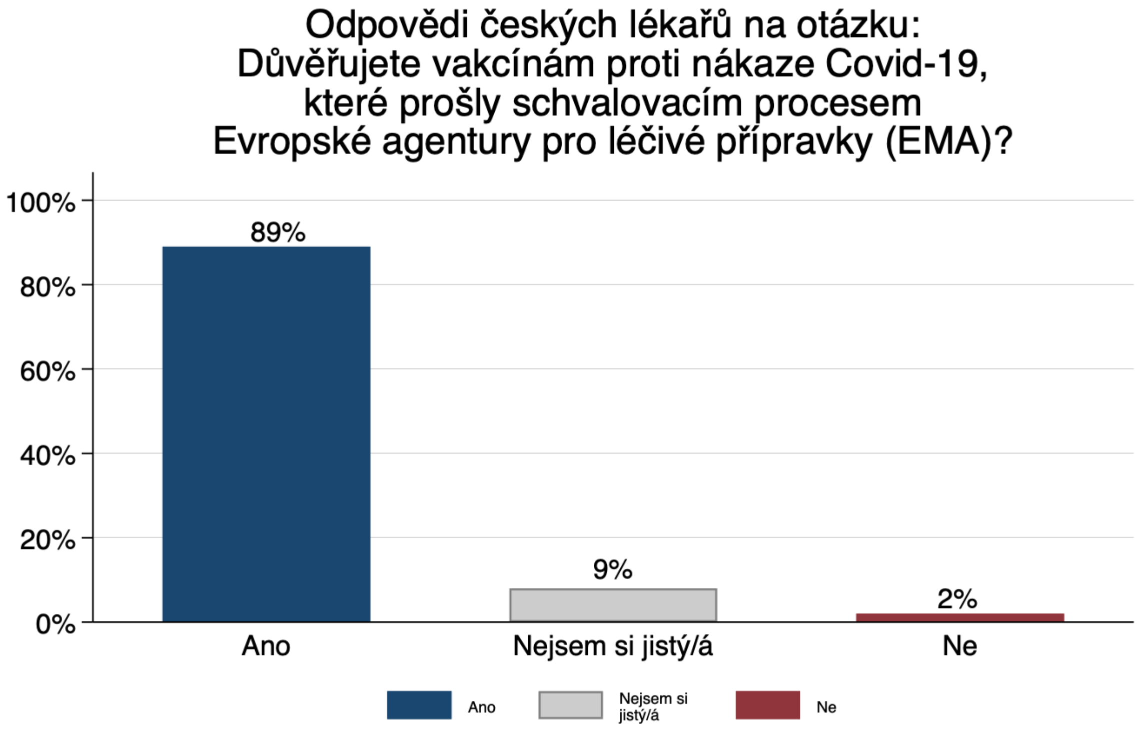

3.4 Verification of vaccination status: Methods and results

The information about vaccination status of the respondents we use in the main analysis is self-reported. In this section, we address a concern that the estimated effect on vaccination take-up may have been affected by experimenter demand effects. Specifically, the concern is that reading about positive views of the medical community about the Covid-19 vaccine might have created a motivation, perhaps to please the survey organizers, to misreport, i.e. to report that a person is vaccinated although s/he is not. Such a pattern of misreporting would lead to an overestimate of the effect of the CONSENSUS condition on genuine vaccine take-up.

We start by noting that the dynamics observed of the effect of the CONSENSUS condition and its persistence are consistent with this explanation only under very specific and rather unlikely assumptions. In general, experimenter demand effects are typically thought to affect survey responses only shortly after the information intervention. This implies that the effect of the CONSENSUS condition should appear already in Wave0 and dissipate over time in later waves. In principle, the effect could persist and show up not only in Wave0 but also later on if participants who misreported their vaccination status in Wave0 remembered their misreports and decided to stick with them in later waves in order to provide consistent answers. However, in contrast, we observe that the effect of the CONSENSUS condition on vaccine take-up does not emerge immediately in Wave0 but emerges only gradually, as more and more people became eligible for the vaccine, and it becomes statistically significant only several months after the information intervention. This pattern could be reconciled with experimenter demand effects only if the respondents remembered feeling compelled to report being vaccinated by the treatment in Wave0, but started strategically misreporting that they got the vaccine only several months later, taking into account changes in the eligibility rules. Furthermore, we show that the treatment increases self-reported intentions to get a booster dose nine months after it was implemented. Although we cannot fully rule out such a persistence of demand effects and high level of sophistication in misreporting, we consider them highly unlikely.

To probe further, we collected additional data in order to empirically verify the vaccination status reported by the respondents in the main survey. We use two different verification methods: (i) Third party verification, and (ii) Certificate verification.

3.4.1. Third-party verification (TPV)

The aim of the Third-party verification survey was to collect comparable data on the vaccination status among the same sample of respondents by another entity, independently from our main data collection. The idea is that when asked by an independent third-party nine-months after the CONSENSUS condition was implemented in a different survey, the experimenter demand effects are unlikely to affect responses.

We took several steps in order to ensure that the respondents perceived TPV as completely unrelated to our main data collection. First, we partnered with a different survey agency (STEM/MARK), which had access to the same panel of respondents. We asked them to incorporate questions on vaccination in one of the surveys sent to respondents on their behalf. Thus, the respondents were approached by a different survey agency, which is seen by the public as a competitor to the one we collaborated with in the main data collection (NMS). Second, the topic of the survey was different. While the topic in our main data collection was life during the pandemic from many perspectives (including changes in employment, financial

situation, mental health), the Third-party verification survey focused specifically on preventive health behavior (including smoking, exercising, preventive visits to the dentist and other specialists, as well as vaccination). Third, the timing of the TPV did not overlap with our main data collection. The Third-party verification survey was launched on December 14, 2021, while the last wave of our main data collection (Wave11) was launched on November 29, 2021 and completed in the first week of December. Finally, the graphical layouts of the survey and the invitation email were also different, reflecting the standards of STEM/MARK.

Sample. Out of the sample of respondents who participated in Wave11 of the main data collection (N = 1,801), 1,672 (92.8%) participated in the TPV. The response rate is similar in the CONTROL condition (92.3%) and in the CONSENSUS condition (93.8%). We matched the data and compared individual reports of vaccination status in Wave11 of the main data collection and in the TPV.

Verification rate. In the TPV, respondents were asked whether they had got at least one dose of Covid-19 vaccine. For those who reported being vaccinated in Wave11 of the main data collection (N = 1,318), we test whether they also reported being vaccinated in the TPV. While 1,316 respondents reported consistently that they were vaccinated in both surveys, only 2 respondents reported being vaccinated in Wave11 of the main data collection and at the same time not being vaccinated in the TPV. Thus, the verification rate of reporting being vaccinated in the main survey is 99.8%. Importantly, the verification rate is not lower in the CONSENSUS condition (99.9%) than in the CONTROL condition (99.8%).[1]

Note that we are unable to verify responses for the whole sample because some people did not participate in Wave11 of the main data collection or in the Third-party verification survey (N = 429; 19.5%). In Section 3.4.3, we show that the effect of the CONSENSUS condition is driven by treated individuals whose reports we can verify in the TPV rather than those whose reports we are unable to verify in the TPV.

3.4.2. Certificate verification (CV)

The second verification aims to link reports of being vaccinated with a proof of vaccination issued by the Ministry of Health of the Czech Republic. It is guided by the idea that people who misreport being vaccinated should not be willing or able to provide information from their administrative records about their vaccination. Thus, in the last wave of the main data collection (Wave11), we aimed to verify the vaccination status of the respondents who reported being vaccinated by asking them to provide information from the official document proving vaccination – the EU Digital COVID certificate. We took advantage of the fact that all vaccinated people in the country received such a certificate and should had it readily available, typically in a mobile app, because in November 2021 there was a legal requirement to screen the certificate in restaurants and other public places.

Sample. We collected the data on vaccination certificates among respondents from our full sample who (i) participated in Wave11 and (ii) reported to have at least one dose of the Covid-19 vaccine in Wave11 (N = 1,414).

[1] Out of those who reported not being vaccinated in Wave11 of the main data collection (N = 354), 336 also reported not being vaccinated in the Third-party verification survey. 18 respondents reported being vaccinated in the TPV. Note that such inconsistency does not necessarily imply misreporting since the TPV took place two weeks after Wave11 of the main data collection and the respondents could have been vaccinated in the meantime.

Verification rates. In the CV, the respondents were asked whether they had their vaccination certificate with them. 1,364 respondents (96.5%) reported to having it and 50 reported not having it readily available. The likelihood of a positive response is very similar across the CONSENSUS (96%) and the CONTROL conditions (97%).

To verify that those who reported to having the vaccination certificate readily available actually had it and did not misreport, we further asked them to copy or type to our survey the text written in two specific text fields in their certificate (Vaccine/Prophylaxis and Vaccine medicinal product). We asked them for this type of information because (i) it does not reveal any personal information, and (ii) the answer is not widely known and it does not include straightforward options, and thus is difficult to guess without seeing the certificate. For example, the text in these parts of the certificate says: SARS-CoV-2 mRNA vaccine, Comirnaty, Spikevax, Vaxzevria, Biontech Manufacturing GmbH. Because there are several types of vaccines and their official names have changed over time, the list of correct answers is not perfectly defined. Further, since participants had to type the answers, a number of them made typos. Thus, in order to evaluate whether each of the respondents most likely had the vaccination certificate or not, we hired two research assistants and asked them to independently evaluate the answers of the respondents, without having access to information about the assignment of respondents to the CONSENSUS or CONTROL condition. They rated each text field by 1 if they were convinced that the respondent saw the certificate when answering the question and by 0 if they thought the respondent did not see the certificate. Since there were two text fields rated by two research assistants, each respondent got four ratings in total. Reassuringly, the ratings of individual raters are highly correlated (for Vaccine/Prophylaxis the raters provided identical ratings in 1331 out of 1364 cases; for the Vaccine medicinal product in 1318 out of 1364 cases). We consider the reports of being vaccinated verified for those respondents who received at least two positive ratings from the research assistants (N = 1,289) and not verified for those who received less than two positive ratings (N = 75) and those who reported not having the certificate with them (N = 50).

The verification rate is 94.4%, conditional on reporting having the certificate. We consider this as relatively high number, given that not being verified does not necessarily imply misreporting, because the respondents might have been vaccinated but did not have the certificate with them, or they had it but did not want to take time to type the text in the survey or they could not read the text in the certificate on their mobile phone screen since it was too small, as some explained in their answers. Thus, for respondents whose vaccination status was not verified, the risk of misreporting might be higher but we cannot precisely estimate the extent of misreporting. That said, importantly, the verification rate is again similar across the CONSENSUS (94.1%) and CONTROL (94.9%) conditions, and thus these findings are not consistent with the concern that the treatment increased misreporting.

In principle, it could still be argued that respondents who chose to misreport their vaccination status could make the effort and find the required pieces of information on the Internet (instead of opting for the simple option of saying that they did not have the certificate with them). To test this rather unlikely possibility, we searched for examples of a certificate ourselves and identified the three first pictures returned by Google search (the search outcomes were the same for different users). All three examples report the same information. Specifically, in the field "Vaccine medical product", the text says "Comirnaty" and in the field "Vaccine/Prophylaxis", the text in internet examples says: "mRNA vakcína proti onemocnění Covid-19. Covid-19 mRNA Vaccine, Severe acute respiratory syndrome coronavirus 2 mRNA only vaccine product (SNOMED CT 1119349007)". As a next step, we identify respondents who provided as their

first answer the word "Comirnaty" and as their second answer at least one of the following four pieces of the text available in the example of a certificate: (i) mRNA vakcína proti onemocnění Covid-19, (ii) Covid-19 mRNA Vaccine, (iii) Severe acute respiratory syndrome coronavirus 2 mRNA only vaccine product, (iv) SNOMED CT 1119349007). Note that these types of answers are expected to be provided by respondents who actually had this type of vaccine and also, potentially, by those who did not have the certificate and found the answer on the Internet. Thus, the prevalence of these types of responses is not informative about the level of misreporting per se. We are again primarily interested in whether it differs across conditions.

Specifically, because there is no reason why the type of vaccine received by the respondents should differ across conditions, greater prevalence of reporting information that matches information in the examples on the Internet in CONSENSUS than in CONTROL could be indicative of greater misreporting of certificate possession. However, this is not what we observe. The prevalence of reporting "Comirnaty" for the first text field is 54.6% in the CONSENSUS condition and 56.3% in the CONTROL condition, the difference not being statistically significant (p-value = 0.541). Similarly, the prevalence of reporting a part of the text field that appears in the example of a certificate for the second question does not significantly differ across conditions - it is 7.1% in the CONSENSUS condition and 7.9% in the CONTROL condition (p-value = 0.561). We conclude that the likelihood of respondents searching for correct answers on the Internet is not higher in the CONSENSUS than in the CONTROL condition and thus, to compare the likelihood of misreporting vaccination status we can rely on comparison of the verification rates based on the raters' assessments reported above.

To summarize, we find that subjects in CONSENSUS, as compared to CONTROL, are (i) not less likely to report having a proof of vaccination readily available, (ii) not less likely to provide specific verifiable information from the certificate, and (iii) not more likely to search for these information on the Internet rather than using their own certificate. Thus, all these results speak against the possibility that subjects in CONSENSUS may be more prone to misreport their vaccination status than those in CONTROL.

In the next sub-section, we show that the effect of the CONSENSUS condition on vaccination status is driven by treated individuals whose vaccination status we can verify based on information provided about their certificate.

3.4.3. Is the effect of the CONSENSUS condition driven by participants whose vaccination status is verified?

As a final step, we explore whether the observed effect of the CONSENSUS condition on vaccine take-up is driven by respondents for whom we can be relatively certain that they were truly vaccinated, rather than mainly by participants for whom we are unable to verify their vaccination status. To do so, we distinguish three categories of answers about vaccination status and estimate the effect of the CONSENSUS condition using the ordered logit analysis (and multinomial logit in a robustness test). In each wave, we classify three types of respondents based on their vaccination status. The first category includes those who reported not being vaccinated and thus could not misreport being vaccinated. We label this category "Not vaccinated" and assign it the lowest value (0), since it is very likely that the respondents were not vaccinated. Next, we classify two categories of respondents who reported being vaccinated. The category "Vaccinated and verified" indicates that the respondent reported being vaccinated and at the same time her/his vaccination status was verified in the TPV/CV.

We assign the highest value to this category (2) since for these respondents it is very likely that they did not misreport their vaccination status and were truly vaccinated. Finally, the category "Vaccinated and not verified" indicates that the respondents reported being vaccinated but their vaccination status was not verified in the TPV/CV, either because their answers in the TPV/CV were evaluated as non-verified or because they did not participate in the TPV/CV. We assign the intermediate value to this category (1) since we are less certain whether they were truly vaccinated.

We use three approaches to use the verification results in the ordered logit analysis. First, we classify the categories based on the results of the TPV. Second, we classify them based on the results of the CV. Third, we classify them using a combination of both verification methods, i.e. we consider as verified those respondents whose reports were verified either by the TPV or by the CV. Further, we provide all these results using two sets of control variables, mimicking the main analysis. First, we control for the pre-specified set of control variables. Second, we control for the variables selected by the LASSO procedure. Since a LASSO command is not available for ordered logit and multinomial logit, we manually add controls selected by the LASSO procedure when analyzing the binary dependent variable (vaccinated vs. not vaccinated).

The results provide a clear pattern. The estimated effect of the CONSENSUS condition on vaccine take-up is almost entirely driven by an increase in the probability of being in the "Vaccinated and verified" group, and not by an effect on the probability of being in the "Vaccinated and not verified" group. We arrive at similar conclusions using both ordered logit and multinomial logit.

Summary

To summarize, we find that large shares of respondents participated in the Third-party verification as well as in the Certificate verification data collections. The rates of verification of the vaccination status among participants are high (91.2-99.8%), suggesting that misreporting is low in general. Importantly, the verification rates are similar in the CONSENSUS condition and in the CONTROL condition for both verification methods, suggesting that misreporting, if any, is not a greater issue in the CONSENSUS condition. Finally, we find that the effect of the CONSENSUS condition on higher vaccine take-up is almost entirely driven by individuals whose reports we can verify. Together, these results boost our confidence in the accuracy of reporting of the vaccination status and attenuate concerns that the effect of the CONSENSUS condition on vaccine take-up could be explained by experimenter demand effects and associated misreporting.

3.5 Regression specification

This section describes the empirical strategy used for the regression analysis.

In our main specifications (Figure 4, Extended Data Table 3), we test the effect of the CONSENSUS condition on vaccination take-up in a given wave (1-11) using two main specifications: (i) a linear probability model (LPM) with a pre-registered set of covariates and (ii) a double-selection LASSO linear regression choosing from a broader set of covariates.

The pre-registered linear probability regression model has the following specification:

$$Y_i = \alpha + \beta CONSENSUS_i + \gamma X_i + \varepsilon_i \quad (1)$$

where Y_i is an indicator equal to 1 if the participant i received at least one dose of a Covid-19 vaccine (Vaccinated; primary outcome, binary).

X_i is a set of individual-specific characteristics and controls. The pre-registered control variables are: gender, age category (6 categories), household size, number of children, region (14 regions), town size (7 categories), education (4 categories), economic status (7 categories), household income (11 categories), and pre-treatment vaccination demand.

The specification of the double-selection LASSO linear regression is equivalent to equation (1). We chose this method on top of the pre-registered LPM because it allows us to identify which covariates have sufficient empirical support for inclusion in the analysis, while tying our hands in the covariate selection process. On top of the pre-registered controls, we let the model select from the following non-specified variables: variable for being vaccinated prior the intervention and prior beliefs about the views of doctors.

A full definition of all variables is provided in Supplementary Information section 3.7. In all models we use Huber-White robust standard errors.

We estimate the models on the full sample of 2,101 respondents who participated in Wave0 (1,940 in Wave1, 1,939 in Wave2, 1,896 in Wave3, 1,860 in Wave4, 1,792 in Wave5, 1,620 in Wave6, 1,770 in Wave7, 1,745 in Wave8, 1,734 in Wave9, 1,611 in Wave10, and 1,851 in Wave11), and the fixed sample of 1,212 respondents who participated across all 12 waves (including Wave-19 on March 1).

In Figure 5 and Extended Data Table 4, we pool data for waves 6-11 when vaccines against Covid-19 were available for all adults and we use either a LPM with a pre-registered set of controls or a double-selection LASSO linear regression. We use the same specification as in equation (1) and add wave fixed effects. We cluster standard errors at the respondent level.

Randomization inference. Beyond standard inference, in Extended Data Tables 3 and 5, we use randomization inference based on permutation tests to construct p-values to test the exact null of no treatment effect[2,3]. We use the Stata package *ritest*[4]. The p-values are computed using 1000 random draws. For pooled regressions, we cluster at the respondent level.

References

1. Komenda M., Bulhart, V., Karolyi, M & et al. Complex reporting of coronavirus disease (COVID-19) epidemic in the Czech Republic: use of interactive web-based application in practice. *J. Med. Internet Res.* **22**, e19367 (2020).
2. Fisher, R. *The Design of Experiments*. (Oliver and Boyd, 1935).
3. Athey, S. & Imbens, G. W. The econometrics of randomized experiments. in *Handbook of economic field experiments* (eds. Banerjee, A. V. & Duflo, E.) 73–140

(North-Holland, 2017).
4. Hess, S. Randomization inference with Stata: A guide and software. *Stata J.* **17**, 630–651 (2017).

3.6 Definitions of variables

Treatment variable

- $\text{CONSENSUS}_i = 1$ if the respondent was randomly assigned to the CONSENSUS condition.

Baseline control variables

- Gender: Female (binary)
- Age category: 18-24 (binary, omitted in regression models to avoid perfect multicollinearity) / 25-34 (binary) / 35-44 (binary) / 45-54 (binary) / 55-64 (binary) / 65+ (binary)
- Household size: "How many members are there in your household?" (integer)
- Number of children: "How many children under 18 or students are there in your household?" (integer)
- Region: Prague (binary, omitted) / Central Bohemia (binary) / South Bohemia (binary) / Plzeň (binary) / Karlovy Vary (binary) / Ústí (binary) / Liberec (binary) / Hradec Králové (binary) / Pardubice (binary) / Vysočina (binary) / South Moravia (binary) / Olomouc (binary) / Zlín (binary) / Moravia-Silesia (binary)
- Town size: Below 999 (binary, omitted) / 1,000-1,999 (binary) / 2,000-4,999 (binary) / 5,000-19,999 (binary) / 20,000-49,999 (binary) / 50,000-99,999 (binary) / 100,000 and above (binary)
- Education: Primary (binary, omitted) / Lower secondary (binary) / Upper secondary (binary) / University (binary)
- Economic status: Answered "What is your economic status?" with: Employee (binary, omitted) / Entrepreneur (binary) / Unemployed (binary) / Retired (binary) / Student (binary) / Parental leave (binary) / Other (binary)
- Household income: Monthly net household income as provided by the Czech National Panel (pre-crisis levels): Up to 10,000 CZK (binary, omitted) / 10,001 – 15,000 CZK (binary) / 15,001 – 20,000 CZK (binary) / 20,001 – 25,000 CZK (binary) / 25,001 – 30,000 CZK (binary) / 30,001 – 35,000 CZK (binary) / 40,001 – 50,000 CZK (binary) / 50,001 – 60,000 CZK (binary) / More than 60,000 CZK (binary) / I don't know (binary) / Missing income data (binary)
- Prior vaccination demand is an indicator variable equal to one if the value for Vaccination demand (see the definition for outcome variables) = 1 in the latest of the six waves of data collection (data collection starting on September 30, 2020, December 8, 2020, January 5, 2021, January 26, 2021, February 16, 2021) for which we have data for a given respondent. In other word, it is the latest pre-treatment indication of vaccination demand for the given respondent (binary).
- Prior vaccination demand missing is an indicator equal to one if we have no record for the previous variable. This was the case for 14 respondents of the full sample of respondents participating in Wave0. In this case of missing data, variable "prior vaccination demand" is coded as 0 (binary).
- Vaccinated at Wave0 is an indicator for whether the respondent received at least one dose of a Covid-19 vaccine by Wave0 (binary).

- Wave0 Beliefs about what percentage of medical doctors plan to get vaccinated (numeric)
- Wave0 Beliefs about what percentage of doctors trust approved Covid-19 vaccines (numeric)

Variables used for sub-sample analyses

In Supplementary Table 11, we conduct the analysis using the model specified in Equation (1) with baseline control variables (defined above) for the following subsamples of respondents i:

- Age: 18-34
- Age: 35-54
- Age: 55+
- Gender: Men
- Gender: Women
- Income: Above median
- Income: Below median
- Town size: Cities
- Town size: Villages/towns
- Education: University
- Education: Completed secondary
- Education: Primary / Not completed secondary
- Demand for information: = 0 if responding [No] to "When it is published, would you like to get a link to a study with the results of a large survey by the Czech Medical Chamber among Czech medical doctors about their attitude to vaccination against Covid-19, which has just been conducted?"
- No demand for information =1 if responding [Yes] to the question above.

4 Populated pre-analysis plan

In the analysis presented above, we closely follow a pre-analysis plan registered at the AEA RCT registry (AEARCTR-0007396; https://www.socialscienceregistry.org/trials/7396).

We only deviate from the pre-analysis plan in the following respects:

- Since the process and requirement of vaccine registrations was changing over time and the vaccine roll-out was in the end faster than expected at the time of pre-registration (March 2021), we decided not to focus on this variable as an outcome of interest. The interpretation of the effects of CONSENSUS on registrations would differ substantially across waves.
- We managed to secure funding for extra survey waves. For this reason, we include eleven waves instead of the pre-registered three, noting that the pre-registration had planned for such a contingency.
- The pre-analysis plan did not plan for the verification methods introduced in Supplementary Information section 3.4.

Next, we present the remaining registered analysis that was not presented in the main text or the supplementary figures and tables. First, we present the results of a survival analysis. Second, we present the heterogeneity of the results by other pre-registered variables, including age, gender, income, town size, education, and demand for information about a full study presenting the results of the Survey among Czech medical doctors.

Supplementary Table 10. Cox proportional hazard model of vaccine take-up by the CONSENSUS condition (Main Experiment)

	(1)	(2)	(3)	(4)	(5)	(6)	(7)	(8)	(9)	(10)
Dependent variable	Vaccinated									
Sample	Full	Full	Fixed	Attentive	Underestimating trust	Overestimating trust	Underestimating take-up	Overestimating take-up	No prior vaccination intentions	Prior vaccination intentions
CONSENSUS	0.084*	0.088*	0.108*	0.084*	0.107**	-0.196	0.112**	-0.555**	0.254**	0.017
	(0.049)	(0.048)	(0.061)	(0.050)	(0.054)	(0.128)	(0.051)	(0.236)	(0.112)	(0.056)
Pre-registered Controls	Yes	No	Yes	Yes	Yes	Yes	Yes	Yes	Yes	Yes
Observations	9,774	9,774	6,198	9,402	9,005	769	9,289	485	5,104	4,670

Notes: Cox proportional hazard model coefficients. Lin and Wei robust standard errors in parentheses. We estimate the hazard of vaccination take-up by the CONSENSUS condition versus the CONTROL condition. Vaccination take-up is an indicator equal to 1 if the respondent reported having obtained at least one dose of a vaccine against Covid-19. Columns 1 and 2 use the full sample. Column 3 uses a sample of respondents participating in all 12 waves. Column 4 restricts the full sample to respondents who passed all attention checks embedded in the survey. Columns 5, 6, 7, and 8 restrict the sample to respondents underestimating doctors' trust in vaccines, overestimating doctors' trust in vaccines, underestimating doctors' intentions to get vaccinated, and overestimating doctors' intentions to get vaccinated, respectively. Columns 9 and 10 restrict the sample to respondents without and with intentions to get vaccinated prior to Wave0, respectively. In Columns 1, and 3-10, we use the pre-registered set of controls. No controls are used in Column 2. Z-test p-values (two-sided) reported as *p<0.10; **p<0.05. No adjustments for multiple comparisons.

Supplementary Table 11. Effect of the CONSENSUS condition on respondents' vaccination take-up: heterogeneity.

	(1)	(2)	(3)	(4)	(5)	(6)
Dependent variable	Vaccinated					
Sample	Full sample			Fixed sample		
**Panel A: Age**	18-34	35-54	55+	18-34	35-54	55+
CONSENSUS	-0.027	0.037	0.039**	-0.038	0.063*	0.052**
	(0.050)	(0.028)	(0.019)	(0.065)	(0.035)	(0.022)
Observations	1,362	3,419	5,501	840	2,280	4,152
CONTROL mean	0.615	0.685	0.804	0.678	0.672	0.795
R-squared	0.352	0.373	0.317	0.456	0.436	0.365
Comparison chi-sq	1.26	0.01	1.57	1.94	0.07	1.80
(p-value)	(0.261)	(0.940)	(0.210)	(0.164)	(0.796)	(0.179)
**Panel B: Gender**	Female	Male		Female	Male	
CONSENSUS	0.033	0.026		0.035	0.061**	
	(0.023)	(0.021)		(0.029)	(0.024)	
Observations	5,161	5,121		3,588	3,684	
CONTROL mean	0.691	0.787		0.695	0.789	
R-squared	0.339	0.340		0.362	0.407	
Comparison chi-sq	0.05			0.49		
(p-value)	(0.823)			(0.485)		
**Panel C: Income**	Below median	Above median		Below median	Above median	
CONSENSUS	0.034	0.026		0.043	0.036	
	(0.029)	(0.019)		(0.034)	(0.024)	
Observations	3,690	5,441		2,604	3,864	
CONTROL mean	0.700	0.782		0.712	0.791	
R-squared	0.309	0.367		0.355	0.384	
Comparison chi-sq	0.04			0.03		
(p-value)	(0.838)			(0.859)		
**Panel D: Town size**	0 - 99,999	100,000+		0 - 99,999	100,000+	
CONSENSUS	0.029	0.051**		0.042	0.071***	
	(0.023)	(0.021)		(0.029)	(0.024)	
Observations	5,248	5,034		3,630	3,642	
CONTROL mean	0.694	0.783		0.690	0.790	
R-squared	0.323	0.353		0.361	0.373	
Comparison chi-sq	0.53			0.62		
(p-value)	(0.468)			(0.430)		
**Panel E: Education**	At most lower secondary	High school	University	At most lower secondary	High school	University
CONSENSUS	-0.002	0.094***	0.016	0.013	0.129***	0.014
	(0.033)	(0.025)	(0.024)	(0.043)	(0.030)	(0.028)
Observations	3,183	3,778	3,321	2,106	2,754	2,412
CONTROL mean	0.683	0.704	0.832	0.689	0.687	0.857
R-squared	0.278	0.410	0.353	0.321	0.466	0.346
Comparison chi-sq	5.54	5.23	0.19	4.94	7.83	0.00
(p-value)	(0.019)	(0.022)	(0.659)	(0.026)	(0.005)	(0.976)
**Panel F: Demand for information**						
	No	Yes		No	Yes	
CONSENSUS	0.046	0.035**		0.085*	0.049**	
	(0.043)	(0.017)		(0.048)	(0.020)	
Observations	1,773	8,509		1,170	6,102	

CONTROL mean	0.690	0.749	0.717	0.748
R-squared	0.352	0.345	0.432	0.380
Comparison chi-sq	0.06		0.50	
(p-value)	(0.808)		(0.481)	

Notes: OLS coefficients. Standard errors clustered at the respondent level in parentheses. The dependent variable in all columns is an indicator for vaccination take-up, equal to 1 if the respondent reported having obtained at least one dose of a vaccine against Covid-19. Wave 6-11 sample used. Columns 1-3 use the full sample. Columns 4-6 use a sample of respondents participating in all 12 waves. In all columns we use the pre-registered set of controls. All columns include wave fixed effects. Panel A presents results split by respondent's age groups. Panel B presents results split by respondent's gender. Panel C presents results split by respondent's income. Panel D presents results split by respondent's town size. Panel E presents results split by respondent's education level. Panel F presents results split by whether the respondent indicated willingness to get information about the survey among medical doctors. Rows titled "Comparison" in report a chi-square statistic and a p-value for a test of equivalence of coefficients across two respective models estimated using seemingly unrelated regressions (suest command in Stata 17). In case of three categories (age and education), we report comparisons for Columns 1 and 2 (4 and 5), Columns 2 and 3 (5 and 6), and Columns 1 and 3 (4 and 6), respectively for full (fixed) samples. T-test p-values (two-sided) reported *p<0.10; **p<0.05; ***p<0.01. No adjustments for multiple comparisons.
