# Supplementary material for: C﻿ommunicating doctors’ consensus persistently increases COVID-19 vaccinations
Source: Nature. 2022 Jun 1;606(7914):542–9. doi: 10.1038/s41586-022-04805-y (PMC9200639; doi:10.1038/s41586-022-04805-y)
Supplement: Supplementary file 2 — Reporting Summary [file 41586_2022_4805_MOESM2_ESM.pdf]

nature portfolio

Corresponding author(s): Vojtěch Bartoš

Last updated by author(s): Apr 5, 2022

Reporting Summary

Nature Portfolio wishes to improve the reproducibility of the work that we publish. This form provides structure for consistency and transparency in reporting. For further information on Nature Portfolio policies, see our Editorial Policies and the Editorial Policy Checklist.

Statistics

For all statistical analyses, confirm that the following items are present in the figure legend, table legend, main text, or Methods section.

n/a	Confirmed	
☐	☒	The exact sample size (n) for each experimental group/condition, given as a discrete number and unit of measurement
☐	☒	A statement on whether measurements were taken from distinct samples or whether the same sample was measured repeatedly
☐	☒	The statistical test(s) used AND whether they are one- or two-sided<br>*Only common tests should be described solely by name; describe more complex techniques in the Methods section.*
☐	☒	A description of all covariates tested
☐	☒	A description of any assumptions or corrections, such as tests of normality and adjustment for multiple comparisons
☐	☒	A full description of the statistical parameters including central tendency (e.g. means) or other basic estimates (e.g. regression coefficient) AND variation (e.g. standard deviation) or associated estimates of uncertainty (e.g. confidence intervals)
☐	☒	For null hypothesis testing, the test statistic (e.g. F, t, r) with confidence intervals, effect sizes, degrees of freedom and P value noted<br>*Give P values as exact values whenever suitable.*
☒	☐	For Bayesian analysis, information on the choice of priors and Markov chain Monte Carlo settings
☒	☐	For hierarchical and complex designs, identification of the appropriate level for tests and full reporting of outcomes
☐	☒	Estimates of effect sizes (e.g. Cohen's d, Pearson's r), indicating how they were calculated

Our web collection on statistics for biologists contains articles on many of the points above.

Software and code

Policy information about availability of computer code

Data collection	Main study / third party verification: CAWI by NMS Market Research (software: NMS CAWI) / STEM/MARK (software iQuest); Survey among medical doctors: Qualtrics; survey distributed by the Czech Medical Chamber
Data analysis	Stata 17; Non-native Stata packages used: ritest (version 2017), orth_out (version 2016), specc (version 2019), grc1leg (version 2014)

For manuscripts utilizing custom algorithms or software that are central to the research but not yet described in published literature, software must be made available to editors and reviewers. We strongly encourage code deposition in a community repository (e.g. GitHub). See the Nature Portfolio guidelines for submitting code & software for further information.

Data

Policy information about availability of data

All manuscripts must include a data availability statement. This statement should provide the following information, where applicable:

- Accession codes, unique identifiers, or web links for publicly available datasets
- A description of any restrictions on data availability
- For clinical datasets or third party data, please ensure that the statement adheres to our policy

The datasets generated during and analyzed during the current study, together with replication files, is available in the Harvard Dataverse repository: https://doi.org/10.7910/DVN/RH0T6R
The availability of the dataset from the Supplementary survey with medical doctors is subject to approval of the Czech Medical Chamber upon request. The reason for not publishing the data is that the doctors were informed that their responses will only be published in an aggregated form to ensure full anonymity. We were aware that some doctors with unique characteristics could potentially be identified from an anonymized but individual-level dataset. Aggregated data can be provided to researchers upon request, additional analysis on an individual level could be requested from the authors.

Field-specific reporting

Please select the one below that is the best fit for your research. If you are not sure, read the appropriate sections before making your selection.

☐ Life sciences ☒ Behavioural & social sciences ☐ Ecological, evolutionary & environmental sciences

For a reference copy of the document with all sections, see nature.com/documents/nr-reporting-summary-flat.pdf

Behavioural & social sciences study design

All studies must disclose on these points even when the disclosure is negative.

Study description	Online experiment (quantitative experimental data) on the effect of providing information about medical doctors' consensus on vaccination demand and vaccination take-up. Accompanying survey among Czech medical doctors provides data for the information treatment in the online experiment.
Research sample	Main experiment (third party verification drawn from the same subject pool as main experiment): Representative sample of the Czech population 18+ in terms of sex, age, education, region, municipality size, employment status before the Covid-19 pandemic, age x sex, and age x education (n = 2,101), sampled from the online panel called "Český národní panel" (Czech national panel). Prague and municipalities above 50,000 are oversampled (boost 200%). The respondents are part of a high-frequency longitudinal study "Life during the pandemic". This allowed us to naturally implement the information intervention and to continue asking questions on vaccination intentions and take-up in a setting familiar to the respondents. The fact that the participants have been participating in up to 24 previous waves of the study prior to the intervention allows us to maintain low rates of attrition and to measure key outcomes repeatedly over an extended period of time. Survey among medical doctors (n = 9,650): survey distributed to all members of the Czech Medical Chamber, response rate 24%. Representativeness discussed in Supplementary Table 1.
Sampling strategy	Main experiment (third party verification drawn from the same subject pool as main experiment): Quota sampling (based on the characteristics specified above) from an online panel called "Český národní panel" (Czech national panel). There are above 1,000 participants in each of the two experimental conditions and thus we are powered to detect even relatively small effects. For vaccination intentions/vaccination take-up in the control group between 0.15-0.75, having a sample size of 1,000 respondents per condition allows us to detect an effect of 0.042-0.056 (alpha=0.05, power=80%, one-sided). Survey among medical doctors: survey distributed to all members of the Czech Medical Chamber
Data collection	Main experiment, third party verification, and Survey among medical doctors: Participants complete the study on their computers or smartphones.
Timing	Main experiment: Wave0: March 15, Wave1: March 29, Wave2: April 12, Wave3: May 3, Wave4: May 24, Wave5: June 21, Wave6: July 19, Wave7: August 23, Wave 8: September 27, Wave 9: October 11, Wave 10: November 8, Wave 11: November 29 (All data collected in 2021; Dates represent the first day of data collection; data collection typically ensued over 5 consecutive days); Survey among medical doctors: February 11-24, 2021; Third party verification: December 15-23, 2021
Data exclusions	Main experiment: we use data for all Wave0 participants (n=2,101); we also report results for a "fixed sample" of participants participating in all eleven waves (n=1,212). Survey among medical doctors: 11,655 respondents opened the survey. Of these, 1,164 answered that they do not currently work in healthcare, 83 workers in healthcare answered that they are not medical doctors and 92 answered that they do not work in the Czech Republic. We excluded these respondents from the analysis. 666 respondents did not complete the survey. In the analysis, we work with sample of 9,650 Czech medical doctors who completed the survey. Third party verification: we use all data for main experiment Wave11 participants (n=1,672). We excluded 50 participants who participated in the Third party verification but not in main experiment Wave11.
Non-participation	Main experiment: The response rate, as compared to the base sample from Wave0, was 92% in Wave1 (March), 92% in Wave2 (April), 90% in Wave3 (May), 89% in Wave4 (May), 85% in Wave5 (June), 77% in Wave6 (July), 84% in Wave7 (August), 83% in Wave 8 (September), 82% in Wave 9 (October), 76% in Wave 10 (early November), and 86% in Wave 11 (late November). Survey among medical doctors: see data exclusions. 666 respondents who satisfy criteria did not complete the survey. Response rate of 24%. Third party verification: 7% of main experiment Wave11 respondents did not participate.
Randomization	Main experiment: Random allocation of participants into CONTROL and CONSENSUS conditions by a computer algorithm. No randomization in the Survey among medical doctors.

Reporting for specific materials, systems and methods

We require information from authors about some types of materials, experimental systems and methods used in many studies. Here, indicate whether each material, system or method listed is relevant to your study. If you are not sure if a list item applies to your research, read the appropriate section before selecting a response.

Materials & experimental systems

n/a	Involved in the study
☒	☐ Antibodies
☒	☐ Eukaryotic cell lines
☒	☐ Palaeontology and archaeology
☒	☐ Animals and other organisms
☐	☒ Human research participants
☒	☐ Clinical data
☒	☐ Dual use research of concern

Methods

n/a	Involved in the study
☒	☐ ChIP-seq
☒	☐ Flow cytometry
☒	☐ MRI-based neuroimaging

Human research participants

Policy information about studies involving human research participants

Population characteristics	See above, Extended Data Table 1 and Supplementary Tables 1 and 3.
Recruitment	Main experiment (third party verification drawn from the same subject pool as main experiment): Members of an online panel "Český národní panel" (Czech national panel) were invited to participate in a survey. Respondents for the panel are recruited by phone calls to randomly generated phone numbers, their identity is cross-validated, they are motivated by financial and non-financial incentives. Internet access is a pre-requisite for participation in our study. Participants were randomized into the experimental conditions. Survey among Czech medical doctors: Members of the Czech Medical Chamber who opted for electronic communication (70%) invited to participate in an online survey. Membership in CMC is mandatory for all Czech medical doctors by law.
Ethics oversight	The research was approved by the Commission for Ethics in Research of the Faculty of Social Sciences of Charles University.

Note that full information on the approval of the study protocol must also be provided in the manuscript.
